# Genome-wide analysis of the passion fruit invertase gene family reveals involvement of *PeCWINV5* in hexose accumulation

**DOI:** 10.1186/s12870-024-05392-y

**Published:** 2024-09-06

**Authors:** Dongmei Huang, Bin Wu, Ge Chen, Wenting Xing, Yi Xu, Funing Ma, Hongli Li, Wenbin Hu, Haijie Huang, Liu Yang, Shun Song

**Affiliations:** 1 Tropical Crops Genetic Resources Institute, National Key Laboratory for Tropical Crop Breeding / Key Laboratory of Crop Gene Resources and Germplasm Enhancement in Southern China, Ministry of Agriculture and Rual Affairs / Key Laboratory of Tropical Crops Germplasm Resources Genetic Improvement and Innovation of Hainan Province / Germplasm Repository of Passiflora, Chinese Academy of Tropical Agricultural Sciences, Haikou, Hainan 571101 P.R. China; 2 Guangxi Crop Genetic Improvement and Biotechnology Laboratory, Key Laboratory of Passion fruit Biology and Genetic Breeding, Ministry of Agriculture and Rural Affairs, Guangxi Academy of Agricultural Sciences, Nanning, Guangxi 530007 P.R. China; 3 Hainan Key Laboratory for Biosafety Monitoring and Molecular Breeding in Off-Season Reproduction Regions, Sanya Research Institute, Chinese Academy of Tropical Agricultural Sciences, Hainan Seed Industry Laboratory, Sanya, Hainan 572025 P.R. China

**Keywords:** Passion fruit, Invertase, Gene family, Abiotic stress, Sugar metabolism

## Abstract

**Background:**

Invertases (INVs) are key enzymes in sugar metabolism, cleaving sucrose into glucose and fructose and playing an important role in plant development and the stress response, however, the INV gene family in passion fruit has not been systematically reported.

**Results:**

In this study, a total of 16 *PeINV* genes were identified from the passion fruit genome and named according to their subcellular location and chromosome position. These include six cell wall invertase (CWINV) genes, two vacuolar invertase (VINV) genes, and eight neutral/alkaline invertase (N/AINV) genes. The gene structures, phylogenetic tree, and cis-acting elements of PeINV gene family were predicted using bioinformatics methods. Results showed that the upstream promoter region of the *PeINV* genes contained various response elements; particularly, *PeVINV2*, *PeN/AINV3*, *PeN/AINV5*, *PeN/AINV6*, *PeN/AINV7*, and *PeN/AINV8* had more response elements. Additionally, the expression profiles of *PeINV* genes under different abiotic stresses (drought, salt, cold temperature, and high temperature) indicated that *PeCWINV5*, *PeCWINV6*, *PeVINV1*, *PeVINV2*, *PeN/AINV2*, *PeN/AINV3*, *PeN/AINV6*, and *PeN/AINV7* responded significantly to these abiotic stresses, which was consistent with cis-acting element prediction results. Sucrose, glucose, and fructose are main soluble components in passion fruit pulp. The contents of total soluble sugar, hexoses, and sweetness index increased significantly at early stages during fruit ripening. Transcriptome data showed that with an increase in fruit development and maturity, the expression levels of *PeCWINV2*, *PeCWINV5*, and *PeN/AINV3* exhibited an up-regulated trend, especially for *PeCWINV5* which showed highest abundance, this correlated with the accumulation of soluble sugar and sweetness index. Transient overexpression results demonstrated that the contents of fructose, glucose and sucrose increased in the pulp of *PeCWINV5* overexpressing fruit. It is speculated that this cell wall invertase gene, *PeCWINV5*, may play an important role in sucrose unloading and hexose accumulation.

**Conclusion:**

In this study, we systematically identified *INV* genes in passion fruit for the first time and further investigated their physicochemical properties, evolution, and expression patterns. Furthermore, we screened out a key candidate gene involved in hexose accumulation. This study lays a foundation for further study on *INV* genes and will be beneficial on the genetic improvement of passion fruit breeding.

**Supplementary Information:**

The online version contains supplementary material available at 10.1186/s12870-024-05392-y.

## Background

Fruits contain a diverse array of sugars, acids, and volatiles, and their chemical compositions play an important role in the perception of fruit flavor [1]. Sugars not only provide “sweetness” as a fruit quality trait but also function as signaling molecules to regulate the accumulation of organic acids, ripening and coloration of fleshy fruit, and modulate the responses of fruit to environmental stimuli [2, 3]. In higher plants, sucrose is synthesized in the leaves and then transported through the phloem to “sink” organs for further metabolism. Sucrose phosphate synthase (SPS), sucrose synthase (SUS), and invertase (INV) are the main enzyme families involved in sugar synthesis and cleavage in sink organs [4]. Among them, INV (EC 3.2.1.26) catalyzes the irreversible hydrolysis of sucrose into glucose and fructose, playing an important role in plant growth and development, stress response, phloem unloading, and source/sink regulation. It is considered a key enzyme in regulating sucrose metabolism [5].

Plant INVs are encoded by a large gene family. Based on their optimum pH levels and subcellular locations, plant INVs can be divided into three subfamilies: cell wall INVs (CWINVs), vacuolar INVs (VINVs), and neutral/alkaline INVs (N/AINVs). CWINVs and VINVs belong to acidic invertases (AINVs) [6]. AINV belongs to the glycoside hydrolase family GH32, which is highly conserved containing N-terminal domain, mature polypeptide, and C-terminal region [5, 7]. CWINVs bind to the cell wall and play essential role in phloem unloading and nonphotosynthetic organs development [7], sugar accumulation and metabolism regulation [8], pests and diseases prevention [9], and fruit growth promotion [10–12]. VINVs are soluble acid invertases located in vacuoles and generally contribute towards sugar accumulation, sucrose/hexose ratio regulation, sugar signaling, cell expansion, and seed development [13–15]. N/AINVs, belonging to the GH100 family known as cytoplasmic invertases (CINs), are exclusively present in plants and photosynthetic bacteria, and they localize within various subcellular compartments including mitochondria, plastids, and nuclei [16]. N/AINVs are non-glycosylated proteins that exhibit characteristic instability but possess greater evolutionary and functional stability compared to CWINVs and VINVs [7]. They have been reported to play roles in root cell development and reproduction [17, 18], as well as sugar homeostasis and stress tolerance in male meiocytes [19]. To date, many INV gene families have been identified in plants, such as Arabidopsis [20], rice [21], sugarcane [22], tomato [6, 7], peach [23], strawberry [24], Chinese white pear [25], and wheat [26]; these studies contribute significantly towards a comprehensive understanding of the structure and function of the INV gene family.

*Passiflora edulis*, commonly known as passion fruit, is a perennial evergreen vine belonging to the Passifloraceae family [27, 28]. Passion fruit is highly favored by consumers in tropical and subtropical regions and has earned the reputation of being the “king of juice”. It is consumed fresh or used as a desirable ingredient in various food products due to its perfect balance of sweetness and acidity, along with its intense aroma and flavor [29]. Among these characteristics, sweetness plays a crucial role in determining passion fruit quality. Sugars constitute the main soluble solids in passion fruit pulp, with sucrose being the predominant sugar followed by glucose and fructose [30, 31]. The composition and concentration of soluble sugars significantly contribute to fruit sweetness and flavor [32]. While previous studies have emphasized the importance of INVs in sucrose metabolism, limited information exists regarding this gene family in passion fruit.

In this study, we identified members of PeINV gene family from the passion fruit genome and conducted an analysis on their phylogenetic relationships, gene structures, protein motifs, and expression patterns during both fruit development and abiotic stress. Furthermore, we identified a key candidate gene associated with sugar metabolism in passion fruit through association analysis between gene expression level and soluble sugar content, and transient overexpression. Our findings will provide valuable insight for further investigations into *INV* gene function and genetic enhancement for passion fruit breeding.

## Results and analysis

### Identification of the INV gene family in *Passiflora edulis*

**Fig. 1:**
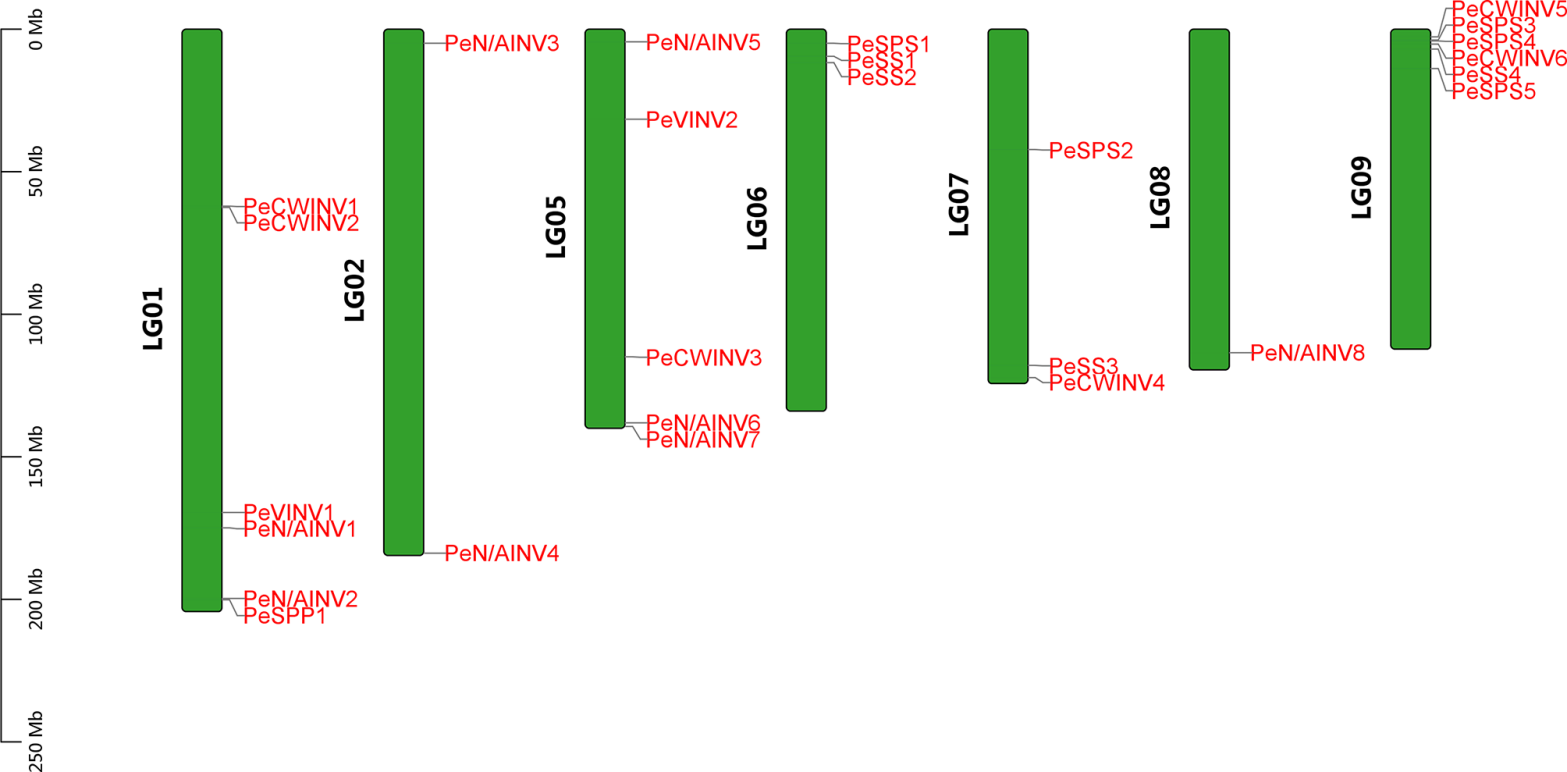
Distribution of the PeINV family on passion fruit chromosomes

A total of 16 INV gene family members were identified from the passion fruit genome using BlastP, HMMER, and CDD searches (Table 1). These genes were distributed on six chromosomes: LG01, LG02, LG05, LG07, LG08, and LG09 (Fig. 1). The amino acid length ranged from 409 aa (PeN/AINV2) to 681 aa (PeN/AINV3), with an average length of 526 aa. The molecular weight varied between 46214.21 u (PeN/AINV2) and 77283.56 u (PeN/AINV3), while the predicted isoelectric point ranged from 4.63 to 9.44. CWINV and VINV contained both conserved Glyco_hydro_32N and Glyco_hydro_32C domains, whereas N/AINV contained the conserved Glyco_hydro_100 domain. All CWINVs and VINVs exhibited stability except for PeCWINV6; however, all PeN/AINVs were found to be unstable proteins. Subcellular location prediction results indicated that six protein are located in the cell wall while two are present in vacuole; eight proteins are localized in the cytoplasm and/or plastids such as chloroplasts and peroxisomes. Consequently, based on their subcellular location and chromosome position, these INV members were named PeCWINV1-PeCWINV6 for those located in the cell wall, PeVINV1-PeVINV2 for those present in vacuoles, and PeN/AINV1-PeN/AINV8 for those localized in cytoplasm and/or plastids regions. PeSUSs, PeSPSs, PeSPP members and more details are shown in Supplementary Table 1.

**Table 1:** Characteristics of 16 PeINV members in *Passiflora edulis*

Gene ID	Gene location	CDS length (bp)	Protein length (aa)	Molecular weight /u	Theoretical pI	Molecular Formula	Subcellular location
PeCWINV1	P_edulis010000882.g	1617	538	60832.99	8.35	C_2760_H_4180_N_748_O_782_S_15_	Cell wall
PeCWINV2	P_edulis010000886.g	1317	438	50135.85	8.89	C_2279_H_3449_N_615_O_645_S_11_	Cell wall
PeCWINV3	P_edulis050012394.g	1386	461	51667.28	5.44	C_2321_H_3551_N_625_O_690_S_13_	Cell wall
PeCWINV4	P_edulis070018529.g	1506	501	55866.63	9.44	C_2517_H_3893_N_691_O_724_S_14_	Cell wall
PeCWINV5	P_edulis090020687.g	1458	485	53498.2	8.46	C_2400_H_3686_N_652_O_719_S_10_	Cell wall
PeCWINV6	P_edulis090021020.g	1734	577	65341.41	5.28	C_2936_H_4448_N_782_O_879_S_18_	Cell wall
PeVINV1	P_edulis010002228.g	1536	511	56870.86	4.63	C_2586_H_3889_N_657_O_773_S_10_	Vacuole
PeVINV2	P_edulis050011950.g	1539	512	57754.96	4.96	C_2611_H_3958_N_680_O_781_S_12_	Vacuole
PeN/AINV1	P_edulis010002781.g	1671	556	63376.85	6.31	C_2847_H_4408_N_762_O_815_S_32_	Chloroplast, Cytoplasmic
PeN/AINV2	P_edulis010005264.g	1230	409	46214.21	5.73	C_2080_H_3223_N_555_O_592_S_23_	Cytoplasmic, Chloroplast
PeN/AINV3	P_edulis020005781.g	2046	681	77283.56	6.46	C_3462_H_5405_N_941_O_1005_S_3_	Chloroplast, Chromoplast
PeN/AINV4	P_edulis020007448.g	1725	574	65806.68	6.34	C_2957_H_4577_N_805_O_836_S_32_	Cytoplasmic
PeN/AINV5	P_edulis050011333.g	1380	459	51830.49	5.4	C_2336_H_3613_N_617_O_672_S_23_	Cytoplasmic
PeN/AINV6	P_edulis050012905.g	1689	562	63558.14	5.19	C_2856_H_4394_N_758_O_846_S_21_	Peroxisomal, Cytoplasmic
PeN/AINV7	P_edulis050012923.g	1737	578	65368.13	5.11	C_2944_H_4510_N_776_O_870_S_21_	Chloroplast
PeN/AINV8	P_edulis080019901.g	1725	574	65592.61	6.3	C_2947_H_4575_N_795_O_835_S_34_	Cytoplasmic

### Phylogenetic relationship analysis of PeINVs

**Fig. 2:**
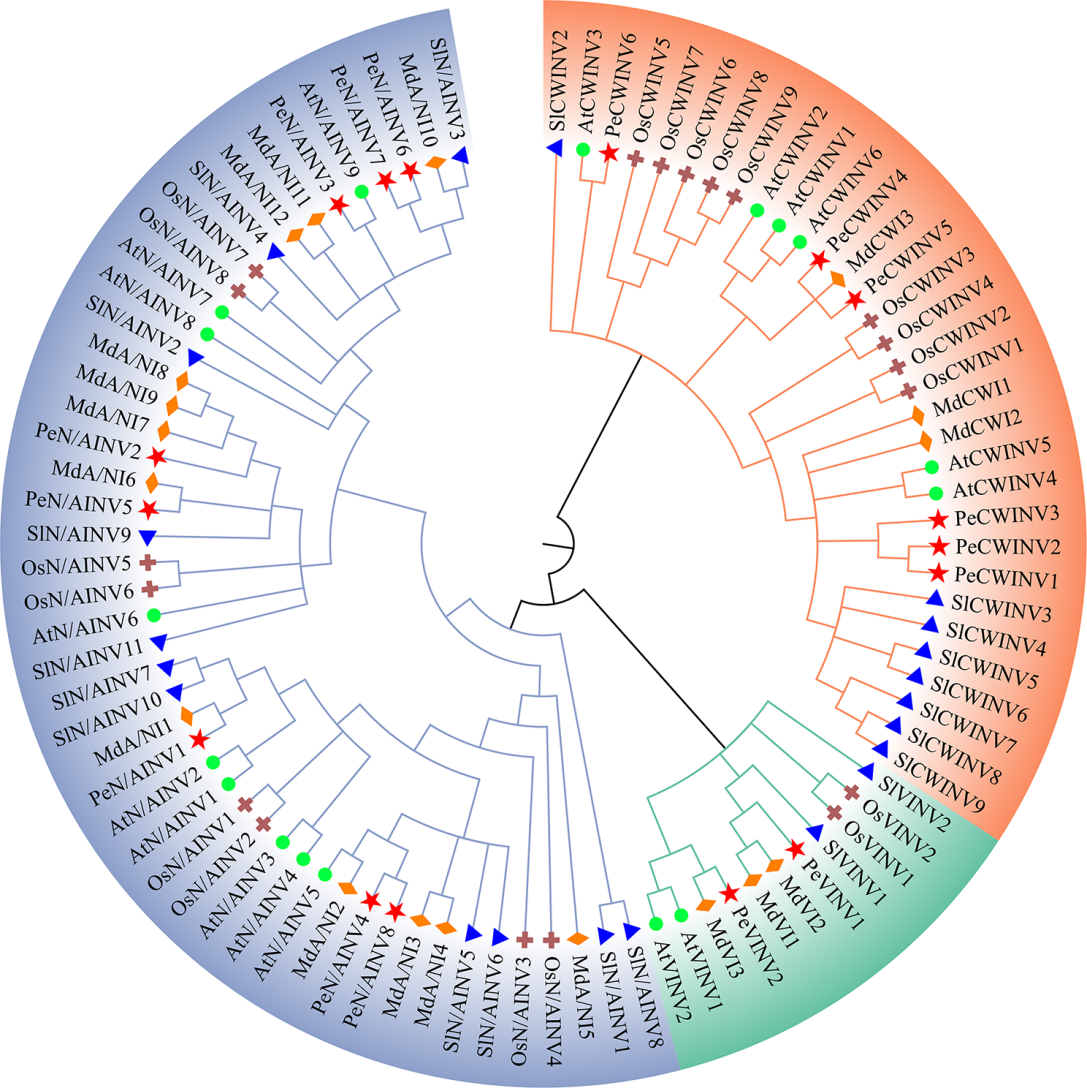
Phylogenetic relationships of INV proteins between passion fruit, Arabidopsis, rice, tomato, and apple. Pe, At, Os, Sl, and Md represent *Passiflora edulis*, *Arabidopsis thaliana*, *Oryza sativa*, *Solanum lycopersicum*, and *Malus domestica*, respectively

In total, 17, 22, 18, and 19 INV members were retrieved from Arabidopsis, tomato, apple, and rice, respectively (Supplementary Table 2). The phylogenetic tree was constructed using the amino acid sequences of these four species along with passion fruit (16 INV members) using the neighbor-joining method (Fig. 2). The results revealed that these 92 proteins can be classified into three groups, N/AINV, CWINV, and VINV, which align with their respective subcellular locations. Amongst the three groups, N/AINV exhibited the highest number of members (8–12 per species), followed by CWINV (3–9 per species), while VINV had the fewest number of members (2–3 per species). In the CWINV group, PeCWINV1, PeCWINV2, and PeCWINV3 showed high homology to AtCWINV4, similarly SlCWINV3 (known as SlLIN5), SlCWINV4 (known as SlLIN7), PeCWINV4, and PeCWINV5 displayed orthologous similarity to AtCWINV2. These findings suggest that these genes may play crucial roles in sugar uptake as well as reproductive organ development such as ovule and fruit development [6, 8, 33, 34]. Within the VINV group, PeVINV1 was closely related to SlINV1 (known as SlVI) and OsINV2 indicating its potential involvement in sugar metabolism [35, 36]. In terms of N/AINV groupings, PeN/AINV1 demonstrated strong orthologous resemblance to AtN/AINV2 (also referred to as AtCIN7, At-A/N-InvG, and AtCYT-INV1) and OsN/AINV1 (also named OsCYT-INV1), whereas PeN/AINV3, PeN/AINV6, and PeN/AINV7 shared close proximity to AtN/AINV7 (also named At-A/N-InvC) and AtN/AINV9 (also named AtCIN1 and At-A/N-InvA), suggesting their association with root cell development, mitochondrial reactive oxygen species homeostasis, and functions in developmental-energy-demanding processes [17, 37–39].

### Gene structure and cis-acting element analysis of PeINVs

**Fig. 3:**
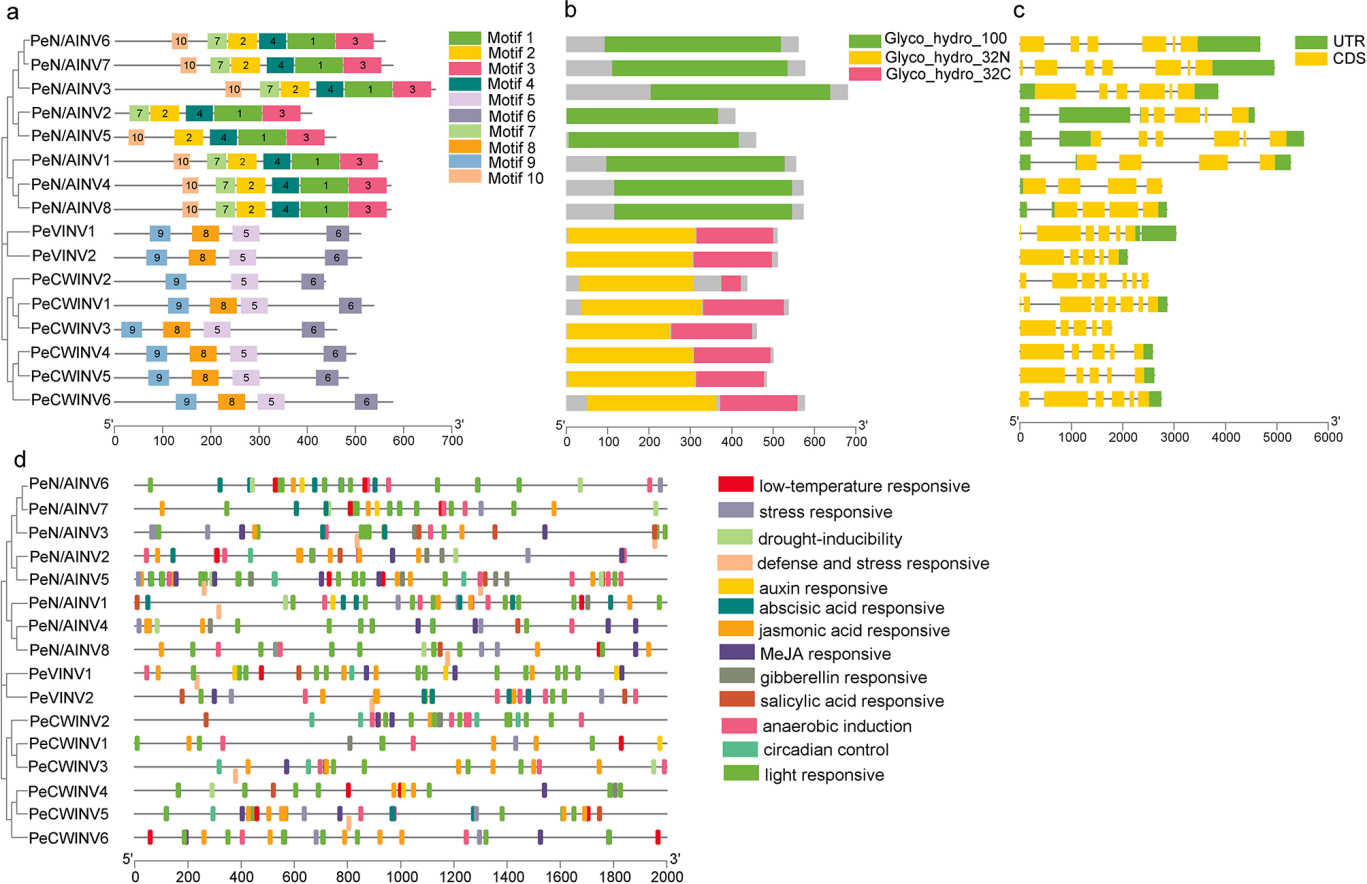
Phylogenetic relationships, gene architecture of conserved protein motifs, gene structures, and cis-elements analysis of PeINVs. (**a**) Motif composition of PeINVs. Different colored boxes display different motifs. (**b**) Conserved domains of PeINVs. (**c**) Exon–intron structure of *PeINV* genes. (**d**) Cis-elements that are related to different stresses and hormones in the 2000 bp promoter upstream of *PeINV* genes

The analysis encompassing phylogenetic relationship, gene structure, and conservative motif will facilitate accurate identification of gene family members and prediction of gene function. The conserved motifs and domains between N/AINVs and AINVs exhibited significant differences. Using MEME software, a total of 10 conserved motifs were predicted in PeINV proteins (Fig. 3a). All N/AINV proteins shared six motifs (motif 1, 2, 3, 4, 7, 10), except for PeN/AINV2, which lacked motif 10, indicating higher conservation in N/AINV protein sequences. Among PeAINV proteins, motif 5, 6, 8, 9 were shared by five PeCWINVs and two PeVINVs; however, PeCWINV2, contained only three motifs (motif 5, 6, 9). Motif 8 contained a conserved catalyze site (WECP/VD) of β-fructosidase while motif 9 contained the conserved β-fructosidase motif “FRDP”. Conservative domain analysis results (Fig. 3b) revealed that N/AINV proteins possessed the conserved Glyco_hydro_100 domain whereas VINV and CWINV proteins had the conserved Glyco_hydro_32N and Glyco_hydro_32C domains, which were consistent with their characteristic [6]. Exon–intron structure analysis results (Fig. 3c) demonstrated diverse numbers of exons in *PeINV* genes ranging from four to eight; however, the structures remained consistent within each INV category suggesting similarity in exon–intron structures within the same group. Notably, somw gene pairs displayed similar exon–intron structures such as *PeN/AINV6*-*PeN/AINV7*, *PeCWINV1*-*PeCWINV2*, and *PeCWINV4*-*PeCWINV5*. This suggested potential functional similarity among these gen pairs.

Furthermore, cis-acting elements present in the predicted promoter regions of *PeINV* genes were investigated (Fig. 3d). Results indicated that enhancers such as TATA box and CAAT box, were found in all PeINVs. Additionally, various environmental or hormone-induced elements including light response (GT1 motif, TCT motif, and G-Box), auxiliary response (TGA element), anaerobic induction element (ARE), abscisic acid response element (ABRE), low-temperature response (LTR) element, stress response element (STRE), MeJA response element (TGACG motif and CGTCA motif), defense and stress response element (TC-rich repeats), and gibberellin response element (P-box, GARE motif, and TATC box), as well as salicylic acid response element (TCA element) were identified. This suggested that *PeINV* genes may be involved in abiotic stress, hormone response, plant growth and development, and other activities. It is worth noting that while *PeN/AINV2*, *PeCWINV1*, and *PeCWINV2* contain fewer response elements (only 2, 6, and 6, respectively), the *PeN/AINV5*, *PeN/AINV6*, *PeVINV2*, *PeN/AINV8*, *PeN/AINV3*, and *PeN/AINV7* promoter regions contain a high amount of responsive elements (49, 33, 28, 28, 27, and 25, respectively).

### Synteny analysis of PeINVs

**Fig. 4:**
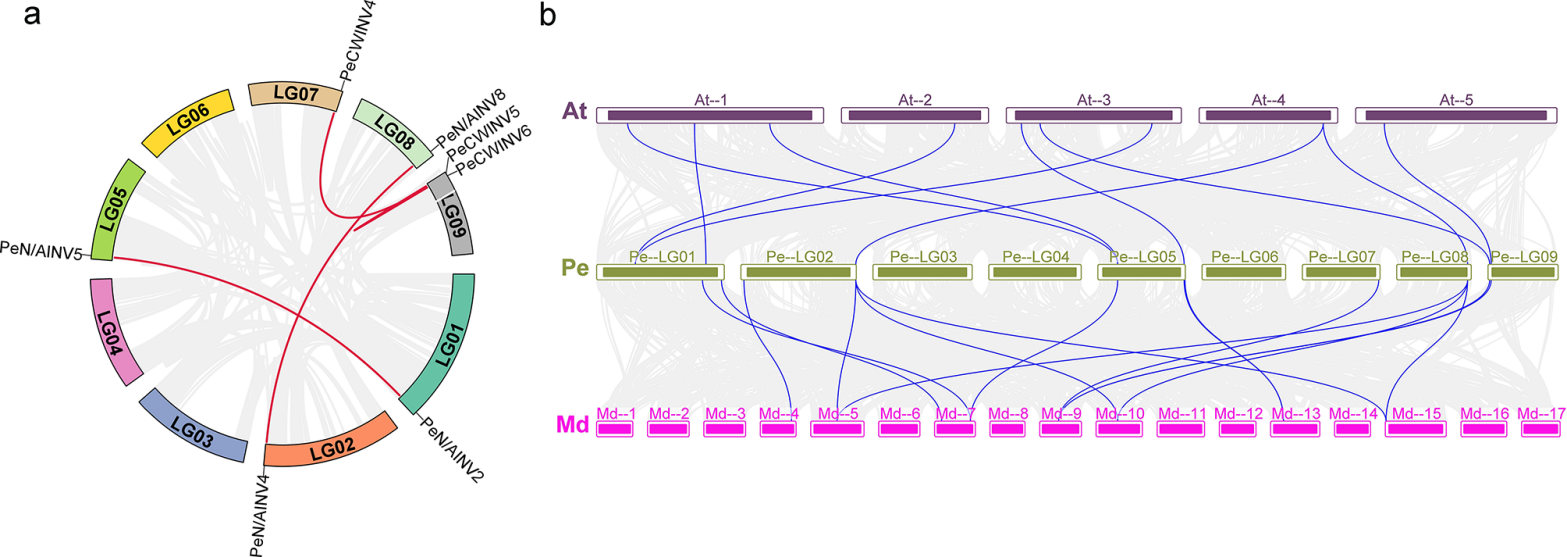
Synteny analysis of INVs in the passion fruit genome (**a**) and among species (**b**). The red line represents the *PeINV* gene pair, and the blue lines represent homologous gene pairs. Pe, At, and Md represent *Passiflora edulis*, *Arabidopsis thaliana*, and *Malus domestica*, respectively

More investigation was done on *PeINV* gene duplication events. It was possible to locate four collinear pairs (Fig. 4a, Supplementary Table 3). One paralogous gene was discovered in two of them, the *PeN/AINV2*-*PeN/AINV5* and *PeN/AINV4*-*PeN/AINV8* gene pairs. It was discovered that *PeCWINV5* shared two paralogous genes (*PeCWINV4* and *PeCWINV6*). The remaining gene pairs were dispersed across several chromosomes, with the exception of the *PeCWINV5*-*PeCWINV6* gene pair on the same chromosome. Events involving gene duplication raised the possibility that these genes would expand and become crucial to evolution.

Furthermore, we investigated the collinearity relationship among INV members in *Passiflora edulis*, *Arabidopsis thaliana*, and *Malus domestica* (Fig. 4b, Supplementary Table 4). A total of eight *PeINV* genes exhibited collinearity relationships with 10 *AtINV* genes and 11 *PeINV* genes showed collinearity with 15 *MdINV* genes. Notebly, both *PeN/AINV4* and *PeN/AINV8* displayed a collinear relationship with one *AtINV* gene (*AtN/AINV5*) and three *MdINV* genes *(MdA/NI1*, *MdA/NI2*, *MdA/NI3*); *PeVINV2* exhibited a collinear relationship with two *AtINV* genes (*AtCWINV1*, *AtCWINV2*) and one *MdINV* gene (*MdVI2*); similarly, *PeCWINV1* demonstrated a collinear relationship with two *AtINV* genes (*AtCWINV4*, *AtCWINV5*). Additionally, we observed that *PeN/AINV6*, *PeCWINV5*, and *PeCWINV6* shared a common collinearity pattern involving one *AtINV* gene pair and one *MdINV* gene: specifically, *PeN/AINV6* was found to be colinear with *AtN/AINV7* and *MdA/NI10*; *PeCWINV5* was colinear with *AtVINV1* and *MdCWI3*; and *PeCWINV6* was colinear with *AtCWINV3* and *MdCWI3*.

### Expression analyses of PeINVs under different abiotic stresses and at different fruit development and ripening stages

**Fig. 5:**
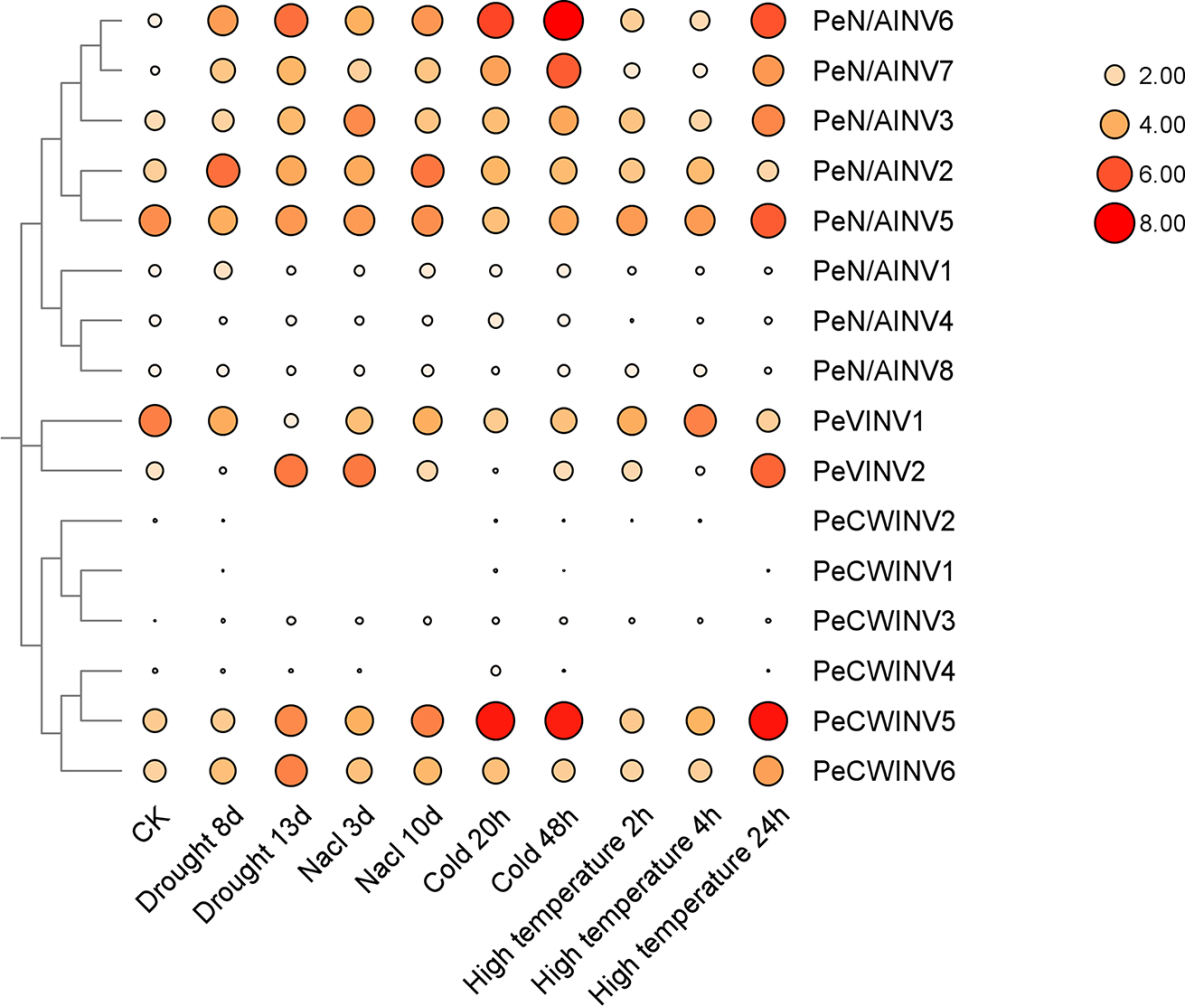
Heatmap of the relative expression of *PeINV* genes responding to drought, salt, cold temperature, and high temperature. White indicates a low expression level, and red indicates a high expression level

The members of the gene family exhibit sequence-level similarity but may vary in terms of their expression levels and functional roles. By analyzing transcriptome and other omics data, it is possible to predict the function of each gene family member and its involvement in growth, development, and adaptation to the environment. In this study, RNA-seq data were utilized to investigate the expression profiles of *PeINV* genes under four abiotic stresses: drought, salt, cold temperature, and high temperature (Fig. 5, Supplementary Table 5). The results revealed differential expression patterns among *PeINV* genes under different abiotic stresses. Notably, *PeCWINV5*, *PeCWINV6*, *PeVINV1*, *PeVINV2*, *PeN/AINV2*, *PeN/AINV3*, *PeN/AINV5*, *PeN/AINV6*, and *PeN/AINV7* exhibited high expression levels under abiotic stresses; whereas the expression level of *PeCWINV1*, *PeCWINV2*, *PeCWINV3*, *PeCWINV4*, *PeN/AINV1*, *PeN/AINV4*, and *PeN/AINV8* were low with minimal changes observed. Specifically, the expressions of *PeCWINV5*, *PeCWINV6*, *PeN/AINV3*, *PeN/AINV6*, and *PeN/AINV7* showed an up-regulated pattern; *PeVINV1* was down-regulated under drought stress; while the expression level of *PeVINV2* was initially decreased followed by an increase with prolong stress time; conversely, *PeN/AINV2* displayed the opposite trend compare to that of *PeVINV2* (first up-regulated then down-regulated). Under salt stress conditions, *PeCWINV5*, *PeCWINV6*, *PeN/AINV2*, *PeN/AINV6*, and *PeN/AINV7* were up-regulated with *PeN/AINV2* showing highest expression level at day10. Under low-temperature stress conditions, *PeCWINV5*, *PeN/AINV3*, *PeN/AINV6*, and *PeN/AINV7* were up-regulated while *PeVINV1* was down-regulated. The expression levels of *PeCWINV5*, *PeCWINV6*, *PeVINV2*, *PeN/AINV3*, *PeN/AINV5*, *PeN/AINV6*, and *PeN/AINV7* did not change significantly at 4 h but increased rapidly at 24 h of high-temperature stress. In particular, the expression level of *PeCWINV5* was the highest at 24 h of high-temperature stress.

**Fig. 6:**
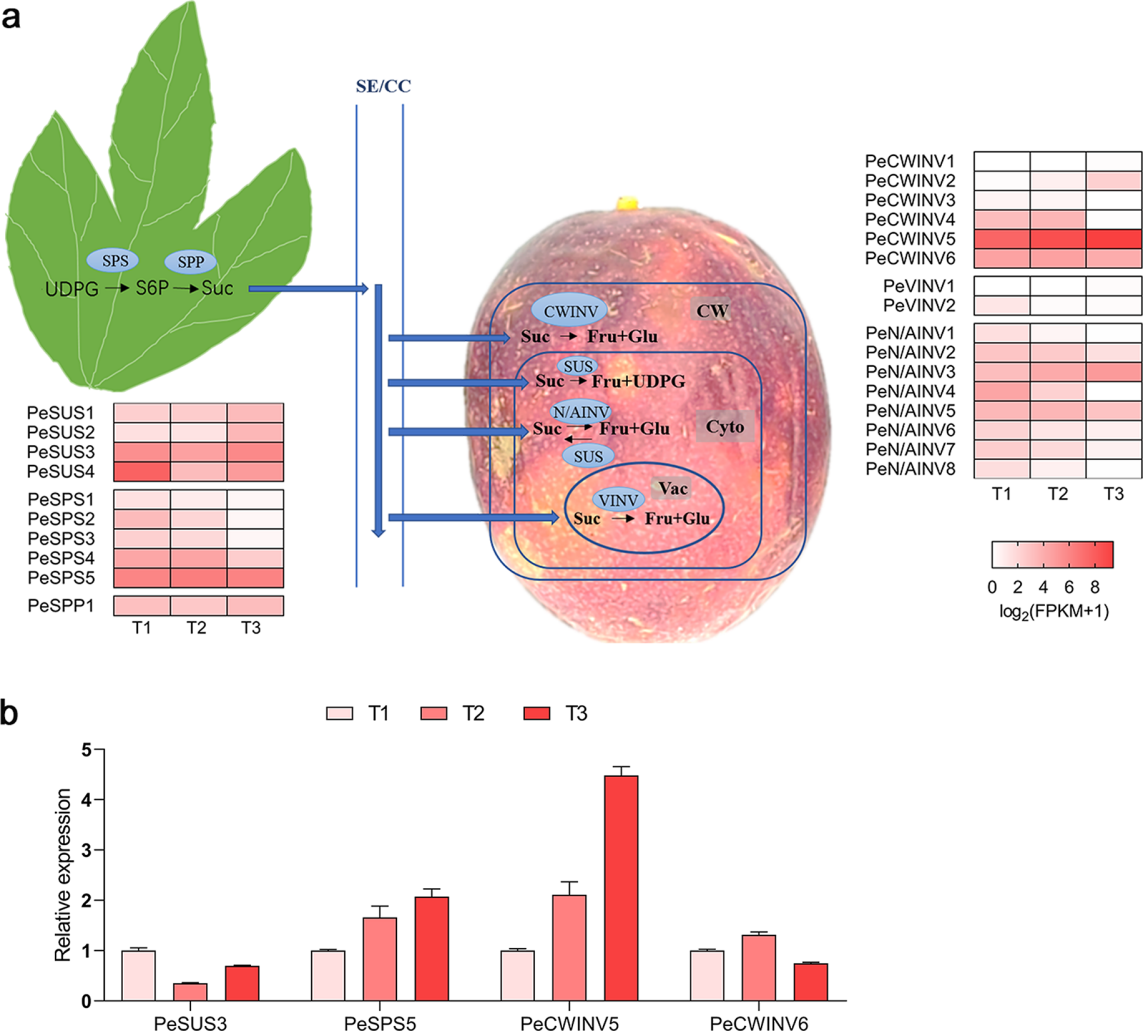
A simplified map of sugar metabolism pathway in passion fruit and heatmap of the expression profile of sugar metabolism genes at three fruit development stages (**a**) and qRT-PCR verification of four candidate genes (**b**). Abbreviation: UDPG, uridine diphosphate-glucose; S6P, sucrose 6-phosphate; Suc, sucrose; Fru, fructose; Glu, glucose; CW, cell wall; Cyto, cytoplasm; Vac, vacuole

The expression levels of sugar metabolism pathway genes, including PeSUSs, PeSPSs, *PeSPP* and PeINVs, were obtained from previous RNA-seq data at three fruit development stages (T1, two weeks before harvest; T2, at harvest time; T3, one week after harvest) (Fig. 6a, Supplementary Table 6) [40]. Differential expression was observed in most genes, with high expression levels (FPKM > 10) detected in *PeSUS2*, *PeSUS3*, *PeSUS4*, *PeSPS4*, *PeSPS5*, *PeCWINV5*, *PeCWINV6*, *PeN/AINV3*, *PeN/AINV4*, and *PeN/AINV5*. During fruit development and ripening progression, down-regulated pattern was observed for several genes including *PeCWINV4*, *PeCWINV6*, *PeVINV2*, *PeN/AINV1*, *PeN/AINV2*, *PeN/AINV4*, *PeN/AINV5*, *PeN/AINV6*, *PeN/AINV7*, *PeN/AINV8*, *PeSUS4*, *PeSPS1*, *PeSPS2*, *PeSPS3*, and *PeSPS4*. Conversely, *PeCWINV2*, *PeCWINV5*, *PeN/AINV3*, *PeSUS1*, and *PeSUS2* showed an up-regulated pattern. Notably, the expression levels of *PeCWINV5* were consistently high and up-regulated throughout all three stages, indicating its pivotal role in passion fruit development and ripening. Furthermore, qRT–PCR validation was performed on four representative members (*PeSUS3*, *PeSPS5*, *PeCWINV5/6*) (Fig. 6b), which confirmed that their gene expressions followed similar patterns as indicated by RNA-Seq analysis.

**Fig. 7:**
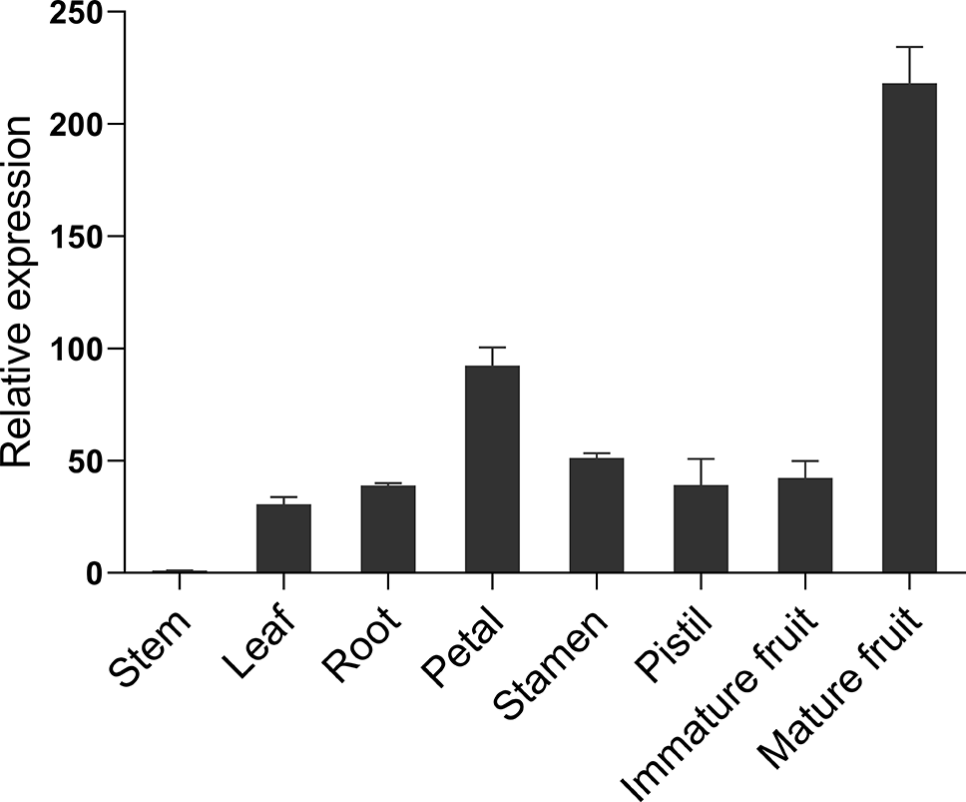
Expression analysis of *PeCWINV5* in different tissues

The expression levels of *PeCWINV5* in eight passion fruit tissues (Fig. 7) were further analzed using quantitative real-time PCR (qRT-PCR). The expression level of *PeCWINV5* was relatively low in nutrient tissues such as roots, stems, and leaves, while it exhibited higher expression levels in flowers and during the development and maturation stages of fruits. Notably, a significant increase in the expression level was observed in mature fruits compared to that at the immature fruit stage.

### Changes in the soluble sugar content during fruit ripening

**Fig. 8:**
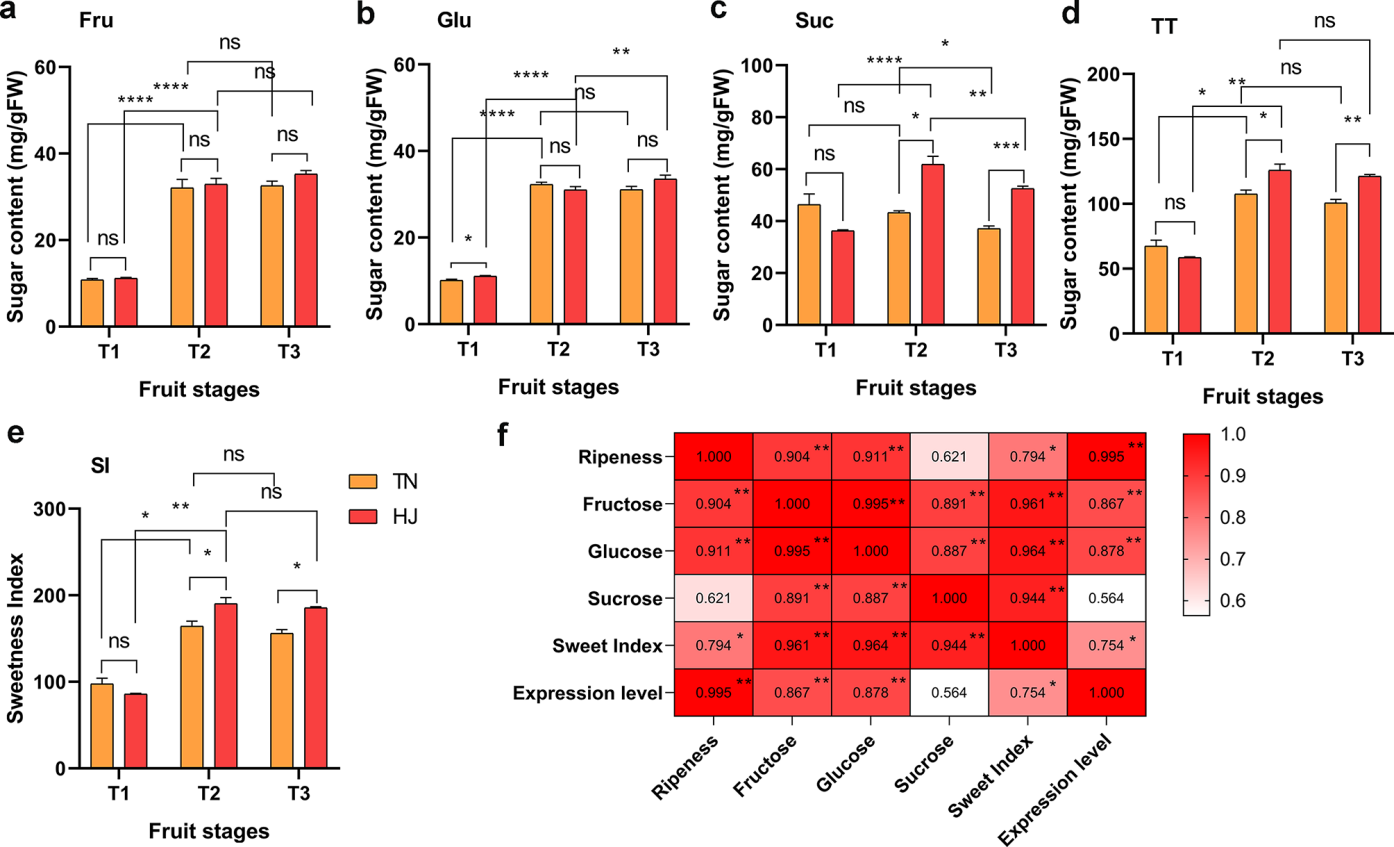
Histogram of the soluble sugar content in the passion fruit varieties “TN” and “HJ” at three stages of fruit development and ripeness. (**a**) Fructose content in “TN” and “HJ”. (**b**). Glucose content in “TN” and “HJ”. (**c**) Sucrose content in “TN” and “HJ”. (**d**) Total sugar content in “TN” and “HJ”. (**e**) Sweetness index in “TN” and “HJ”. (**f**) Pearson correlation analysis of fruit ripeness, sugar content, sweetness index, and relative expression of *PeCWINV5* in variety “TN”. TT and SI represent total sugar content and sweetness index, respectively. ns, *, **, ***, **** represent no significant, significant at *p* < 0.1, *p* < 0.01, *p* < 0.001 level, respectively

The changes in soluble sugar content during fruit ripening of “TN” and “HJ” varieties were analyzed using high-performance liquid chromatography (HPLC) (Supplementary Table 7). The soluble sugar components identified in both varieties of passion fruit included sucrose (Fig. 8a), glucose (Fig. 8b), and fructose (Fig. 8c). Furthermore, the total sugar contents (Fig. 8d) and sweetness index (Fig. 8e) were assessed for both varieties. In the case of “TN,” the sucrose content remained relatively stable throughout development and maturity, while the levels of fructose and glucose significantly increased from T1 to T2 by approximately 2.96- and 3.18-fold, respectively; however, no significant changes were observed from T2 to T3. The contents of fructose and glucose exhibited similar patterns across all three stages. Consequently, the total soluble sugar content increased due to accumulation of fructose and glucose from T1 to T2, resulting in an increase in sweetness index from 97.67 at T1 to 164.46 at T2; thereafter, there was no noticeable change between T2 and T3 stages for these parameters.

Similar trends were observed for “HJ”, with a significant increase in fructose (approximately 2.94-fold) and glucose (approximately 2.79-fold) levels fromT1 toT2; however, no substantial changes occurred between T2 and T3. The trend for sucrose accumulation differed compared to that observed for “TN”. Sucrose content rapidly increased by approximately 1.7 times from T1 to T2 and then decreased from T2 to T3. The overall dynamics of total soluble sugar content reflected variations in fructose, glucose, and sucrose accumulations. Sweetness index values were initially recorded as 85.82 at T1, increased rapidly to 190.35 at T2, and subsequently decreased to 185.55 at T3, which was higher than that observed for “TN”.

The Pearson correlation analysis was performed to investigate the relationship between fruit ripeness, sugar content, sweetness index, and *PeCWINV5* expression levels during the three stages of development and ripening in “TN” (Fig. 8f, Supplementary Table 8). The obtained correlation coefficients were equal to or greater than 0.564, indicating strong positive correlation. Notably, the expression level of *PeCWINV5* exhibited a consistent increasing trend along with fructose content, glucose content, and sweetness index during fruit development and ripening stages; these variables showed significant correlations with each other. Moreover, there were also highly significant correlations observed among sucrose content, fructose content, glucose content, and sweetness index. Based on these findings, we conclude that as fruit ripeness increases in “TN”, the upregulation of *PeCWINV5* expression promotes hexose synthesis leading to an increase in fruit pulp’s sweetness index.

### Transient overexpression in passion fruit pulp

**Fig. 9:**
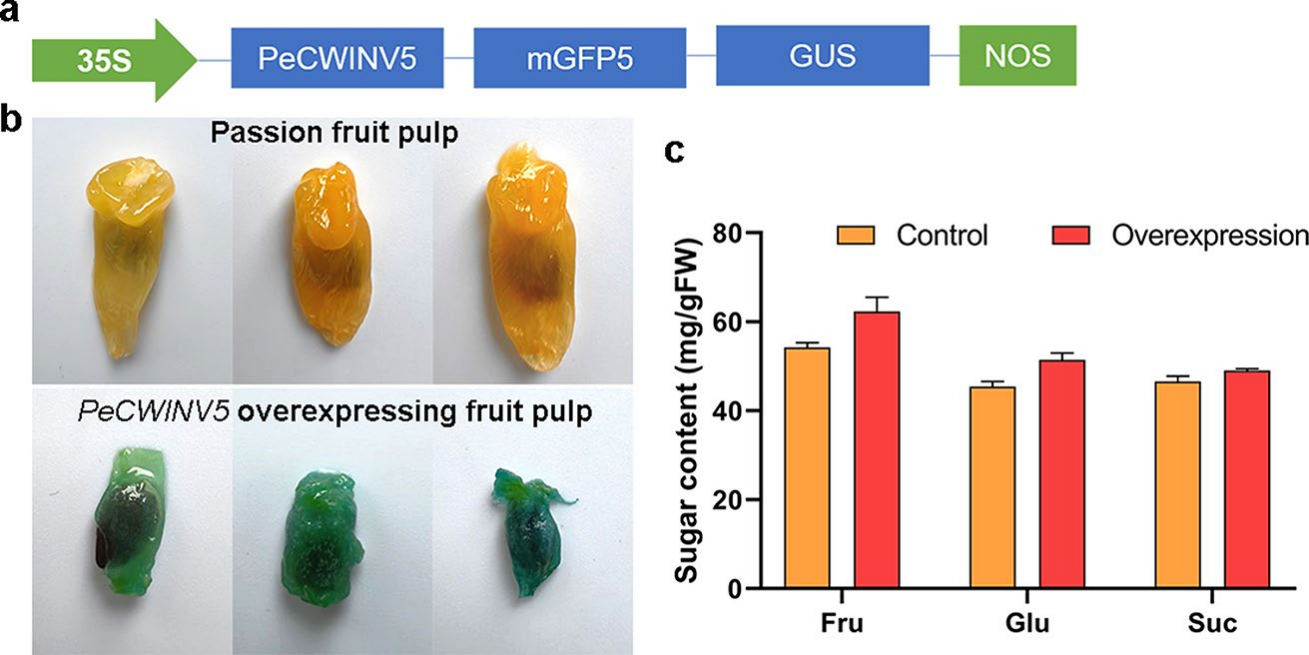
Transient overexpression in passion fruit pulp. (**a**) Schematic representation of pCAMBIA1304-*PeCWINV5* plasmid. (**b**) Passion fruit pulp and histochemical GUS staining of passion fruit pulp after injection with Agrobacterium to obtain transient expression of *PeCWINV5*. (**c**) Effects of transiently overexpressed *PeCWINV5* on the sugar content after injection with Agrobacterium. Fru, Glu, and Suc represent fructose, glucose, and sucrose, respectively

The pCAMBIA1304-*PeCWINV5* overexpression construct was introduced into passion fruit pulp through Agrobacterium tumefaciens mediated injection (Fig. 9a). As shown in Fig. 9b, GUS staining results demonstrated the successful transfer and overexpression of pCAMBIA1304-*PeCWINV5* in passion fruit. Following a three-day co-culture period, the treated pulp was selected for sugar content evaluation. Notably, compared to the control group, fructose, glucose, and overall sugar content exhibited significant increases in passion fruit pulp overexpressing *PeCWINV5* (Fig. 9c).

## Discussion

### INV play a crucial role in regulating plant growth and sucrose metabolism

In plants, sucrose is the final product of photosynthesis and can be transported to non-photosynthetic tissues through the phloem. It undergoes enzymatic degradation by INVs into fructose and glucose, which provide energy and support growth in sink organs [6]. Sugar plays a crucial role in various plant development and metabolic processes, as well as influencing nutrient production, flavor, odor, and response to biological and abiotic stresses [2]. In passion fruit, soluble sugars primarily consist of sucrose, fructose, and glucose. The soluble sugars in passion fruit undergo dynamic changes during fruit development and ripening. Sucrose content remains stable or slightly increases throughout this process. However, fructose and glucose contents significantly increase during early fruit development while maintaining a balance in the late ripening stage. This suggests that INV plays a crucial role in hexose accumulation during fruit development and ripening. In this study, we conducted a genome-wide analysis of the PeINVs family. Through gene structure and phylogenetic relationship analysis, we identified six *PeCWINV* genes, two *PeVINV* genes, and eight *PeA/NINV* genes.

The INV family can be categorized into acid invertases and neutral invertases, displaying limited sequence similarity and divergences in terms of optimal pH, subcellular localization, and functionality [41]. Acid invertases comprise CWINVs and VINVs belonging to the GH32 family. CWINVs are localized in the cell wall and have coevolved with the vasculature, playing crucial role in plant fruit growth, pest defense, and phloem unloading [7]. *CWINV* is essential for fruit and seed setting [42]. In Arabidopsis thaliana, *CWIN2* and *CWIN4* couple with hexose transporters to promote ovule initiation [34]. Lycopersicum invertase 5 (*LIN5*) in tomato is associated with pollen tube growth. It increases activity during the transition from ovary to fruit, regulates cell division and thus fruit setting, and increases the fruit soluble content [33, 43]. *TaCWINV40* and/or its highly similar replicate genes are involved in the anthers development as well as pollen development [26]. CWIN activity is induced or enhanced during pathogen infection, and it enhances the sink strength of infected tissues and potentiates the local defense at the infection site [9]. VINV is located in vacuoles. Its function is more reflected in sugar accumulation and osmotic regulation by regulating the sucrose/hexose ratio which affects fruit quality such as color and sweetness [3, 7]. In tomato, *SlVI* interacted with *SlVIF* (invertase inhibitor) to control sucrose metabolism, thereby affecting the production of ethylene and fruit ripening [35]. *OsVIN2* regulated glucose metabolism and is essential for rice grain size, formation, and development [36]. The overexpression of *CsINV5* enhanced plant cold tolerance by modifying the content of cellular sugar compounds and osmotic-regulation-related pathways [44]. *IbVIN1* plays a central role in the manipulation of VIN activity and consequently regulates the reducing sugar content in postharvest sweet potato [15]. It is worth noting that among the INV family members of the reported species, the number of VINVs is the lowest, stable at 2–3. Therefore, it can be assumed that VINV remains relatively stable during evolution. The N/AINVs are members of the glycoside hydrolase 100 (GH100) enzyme family, which are enzymes found in oxygenic photosynthetic organisms and localized in the cytoplasm or organelles [16]. Despite their poor stability and low activity, it is noteworthy that the N/AINV family has the largest number of members, with segmental duplication contributing to its expansion [45]. In Arabidopsis, apple, rice, and passion fruit, the proportion of N/AINVs is 9 out of 17, 12 out of 18, 8 out of 19, and 8 out of 16, respectively. This high proportion suggests that N/AINVs may play a crucial role in plant growth and development. For instance, *AtCIN7* (*AtA/NINV2*) serves as a key enzyme regulating the concentration levels of sucrose, glucose, and frutose in the cytoplasm [46] and participates in osmotic stress-induced lateral root growth by modulating intracellular hexose concentration [37]. *AtA/NInvA* (*AtA/NINV9*), *AtA/NInvC*, and *AtA/NInvG* (*AtA/NINV2*) are capable of producing glucose as a substrate for mitochondria-associated hexokinase to promote plant growth and development [38, 39]. *OsCYT-INV1* has been reported to play a pivotal role in plant morphology, growth, and development [17]. A/NINV activity in wheat is associated with efficient hydrolysis of intracytoplasmic sucrose under stress conditions [47]. N/AINV also significantly contributes to the regulation of reducing sugars and the process of cold-induced sweetening in potato [48]. In conclusion, all three types of INVs play important roles in plant growth and sugar metabolism, thereby offering valuable insights for further investigation and functional analysis of INV family genes in passion fruit.

### The expression of *INV* genes are affected by hormones and abiotic stresses

Invertase plays an important role in the determining plant growth and quality characteristics, and the expression of genes is influenced by many factors. In apple, water-stress treatment leaded to higher enzymatic activity of VINV throughout fruit development [49]. *SoCIN1* expression was induced by 15% PEG, low temperature, and salt stress in both leaves and roots of sugarcane [50]. Cold stress suppressed the transcription of *INVINH1* (a cell wall invertase inhibitor) while increasing Lin6 and Lin8 (cell wall invertase genes) expression in tomato seedlings [51]. Most of the *GhN/AINV* genes were induced in response to PEG or salt stress [52]. The transcription level of the *CsINV5* gene increased with decreasing temperature in tea plants, and its overexpression enhanced cold tolerance in transgenic Arabidopsis [44]. Exogenous ABA, GA3, MeJA, and high temperature all induced the expression of *MeCWINV3* in cassava seedlings [53]. Chilling temperature affected *PpVIN2* sensitivity and sucrose metabolism regulation in postharvest peach fruit [23], where it interacted with *PpINH1* for chilling tolerance regulation as well [54]. GA had an inducible effect on *VvGIN1* and *VvGIN2* promoters during fruit setting process and increased the sink strength and sugar signaling activity [55]. In this study, cis-acting element prediction analysis revealed that the upstream promoter region of *PeINV* genes contains response elements for low temperature, defense and stress, as well as MeJA, ABA, salicylic acid, and GA3. Notably, *PeVINV2*, *PeN/AINV3*, *PeN/AINV5*, *PeN/AINV6*, *PeN/AINV7*, and *PeN/AINV8* promoter regions exhibit a higher abundance of these response elements. Therefore, it can be speculated that *N/AINV* gene expression is significantly influenced by the external environmental condition, which is consistent with their instability characteristic. In addition, expression patterns under different abiotic stresses were investigated, revealing that *PeCWINV5*, *PeCWINV6*, *PeVINV1*, *PeVINV2*, *PeN/AINV2*, *PeN/AINV3*, *PeN/AINV6*, and *PeN/AINV7* responded significantly to drought, salt, and high-temperature stresses. These findings are consistent with the results of cis-acting element prediction.

### *PeCWINV5* is involved in hexose accumulation in passion fruit

We further investigated the role of *PeINV* genes in the regulation sugar accumulation in the fruit, which is considered as the primary sink organ. In peach, the up-regulation of *PpVAINV2* leads to sucrose cleavage, and sucrose content initially increased and then decreased during fruit ripening, while glucose and fructose exhibit an opposite pattern [56]. During cold storage, post-translational repression of VIN enzyme activity by *PpINH1* helped maintain sucrose levels and enhanced resistance against chilling injury [54]. The ratio of sucrose to hexose plays a crucial role in fruit maturity regulation and is modulated by INV and SPS activities [14]. Moreover, VIN activity and sweetness index are correlated with fructose/glucose content [15]. In ‘Dangshansuli’ pear development, changes in invertase activity along with expression profiles of *PbrInvs* were associated with visual/inner quality alterations. Additionally, it is suggested that *PbrvacInv1* might participate in sucrose decomposition during pear development [25]. Furthermore, there existed a significant correlation between total sugar content in strawberries and the expression level of *FaCWINV1*. Notably, *FaCWINV1* exhibited significantly up-regulated expression during fruit development and strong expression in mature fruits, indicating its potential involvement in sugar accumulation within strawberries [24]. *HpVAI1* played a key role in in the regulation of glucose metabolism throughout dragon fruit development [57].

In passion fruit, the sucrose content remained relatively stable during fruit maturity, while the fructose and glucose contents exhibited a rapid increase, indicating that invertase plays a crucial role in cleaving most of the newly transported or resynthesized sucrose into fructose and glucose. Therefore, we investigated the transcript profiles of sugar metabolism pathway genes at three different stages of passion fruit development. SUS in plants is involved in regulating sink intensity as well as phloem loading and unloading by catalyzing both sucrose synthesis and decomposition [58]. SPS irreversibly catalyzes the reaction between fructose-6-phosphate (F6P) and uridine diphosphate glucose (UDPG) in sucrose metabolism to produce sucrose-6-phosphate (S6P), which is further converted to sucrose by SPP [59]. Our findings revealed high expression levels (FPKM > 10) of *PeSUS2*, *PeSUS3*, *PeSUS4*, *PeSPS4*, *PeSPS5*, *PeCWINV5*, *PeCWINV6*, *PeN/AINV3*, *PeN/AINV4*, and *PeN/AINV5* during fruit development stages, suggesting their potential essential roles in sugar metabolism or other processes. The hexose content comprising glucose and fructose significantly increased during passion fruit ripening with INV playing a key role in hexose synthesis. Henceforth, the focus was on examining the expression pattern of *INV* genes. The up-regulated trend was observed for *PeCWINV2*, *PeCWINV5*, and *PeN/AINV3*, with *PeCWINV5* being the most abundantly expressed at ripening stages, suggesting its involvement in sugar accumulation and contribution to the increase in the sweetness index of passion fruit. Relative expression of *PeCWINV5* in eight tissues indicated its high expression level in mature fruits and flower organs, which are associated with fruit setting and ripening. Correlation analysis conformed the significant correlation among fruit ripeness, soluble sugar content, sweetness index, and *PeCWINV5* expression level. Considering the optimization required for the passion fruit transformation system, which takes 1–2 years to generate transgenic fruit, we employed the Agrobacterium transient transformation system to investigate the biological functionality of *PeCWINV5*. Our findings demonstrate that *PeCWINV5* overexpression in fruit pulp leads to an elevation in fructose, glucose, and sucrose levels. These results suggested that *PeCWINV5* not only contributes to hexose accumulation but may also facilitates sucrose transport and unloading in sink organs such as fruits.

## Conclusions

In this study, we identified a total of 16 *PeINV* genes based on the whole-genome data of passion fruit and conducted comprehensive bioinformatics analyses to investigate their physicochemical properties, chromosome location, phylogenetic relationships, structural features, cis-acting elements, and synteny. Furthermore, we performed expression profile analysis using transcriptome data under four abiotic stresses and at three fruit development and ripening stages to elucidate the functions of *PeINV* genes. Additionally, through association analysis of *PeCWINV5* expression and soluble sugar content as well as transient overexpression analysis, we successfully identified a key candidate gene involved in sucrose unloading and hexose accumulation. Our findings provide valuable insights for further exploring the functions of INVs and facilitating genetic improvement in passion fruit breeding.

### Methods

#### Identification of the INV gene family in passion fruit

The genome data of purple passion fruit (*Passiflora edulis* sims) were obtained from the National Genomics Data Center (https://ngdc.cncb.ac.cn/search/?dbId=gwh&q=GWHAZTM00000000). A local BlastP search using 17 protein sequences of AtINVs downloaded from The Arabidopsis Information Resource (TAIR, https://www.arabidopsis.org/) was performed against the passion fruit genome with NCBI-Blast + version 2.11.0, employing an E-value threshold of 10^− 5^ and identity cutoff of > 50%. Additionally, an HMMER search was conducted utilizing the INV protein domain (PF00251 glycosyl hydrolase family 32 N-terminal domain, PF08244 glycosyl hydrolase family 32 C-terminal domain, PF12899 alkaline and neutral invertase) from the PFAM database (http://pfam.xfam.org/) as a template with E-value = 10^− 5^. For identification of SUS, SPS and SPP proteins, HMM searches were carried out using SUS domain (PF00862 Sucrose-synth, PF00534 Glycos-transf-1), SPS domain (PF00862 Sucrose-synth, PF00534 Glycos-transf-1, PF05116 S6PP), SPP domain (PF05116 S6PP, PF08472 S6PP_C). The retrieved protein sequences were further filtered and confirmed using the online Conserved Domains search tool (National Center for Biotechnology Information; http://www.ncbi.nlm.nih.gov/Structure/bwrpsb/bwrpsb.cgi) with default parameters; only genes containing the conserved domain were considered members of their respective gene families. In total, we identified 16 INV protein, four SUS protein, five SPS protein, and one SPP protein sequence for subsequent analysis. Physical and chemical parameters of these proteins were computed using EXPASY online tool (https://web.expasy.org/protparam/), while subcellular localization prediction was performed using WoLF PSORT (GenScript; https://www.genscript.com/wolf-psort.html). Chromosome localization visualization for these genes was achived through TBtools software [60].

### Phylogenetic relationship, conserved motif, gene structure, and cis-acting element analyses

For phylogenetic relationship analysis, the protein sequences of SlINV, MdINV, and OsINV were retrieved from the genome databases of tomato (*Solanum lycopersicum*; https://solgenomics.net/organism/Solanum_lycopersicum/genome, ITAG4.1), apple (*Malus domestica*; GDR, https://www.rosaceae.org/, v3.0.a1) [61], and rice (*Oryza sativa*; http://rice.uga.edu/, version_7.0). Multiple sequence alignment analysis was performed using MEGA software (version X) to construct a neighbor-joining phylogenetic tree including passion fruit, *Arabidopsis thaliana*, apple, tomato, and rice INV proteins. The pairwise deletion option and Poisson correction model with 1000 bootstrap replicates were employed as criteria for tree construction. ChiPlot (https://www.chiplot.online/) was utilized for enhancing the visual presentation of the phylogenetic tree.

The MEME (Multiple EM for Motif Elicitation; http://meme-suite.org/) program was utilized for motif annotation, with the number of motifs set to 10 and the maximum width set to 100. Default values were used for the remaining parameters. Conservative structure prediction was performed using the batch CD search domain (https://www.ncbi.nlm.nih.gov/Structure/bwrpsb/bwrpsb.cgi). The upstream 2000 bp genomic DNA sequences from each P*eINV* gene’s transcriptional start site were filtered and uploaded to the online PlantCARE database (BioTools; http://bioinformatics.psb.ugent.be/webtools/plantcare/htmL/) to identify cis-acting elements, while retaining induction and response factors. All results were visualized using TBtools software. Gene duplication events within and between species were analyzed using Multicollinearity Scanning Toolkit (MCScanX) and TBtools software.

### Expression analysis of PeINVs

The transcriptomic data of PeINVs in response to four abiotic stresses (drought, salt, cold temperature, and high temperature) [62], as well as the expression profiles of sugar metabolism pathway genes including PeSUSs, PeSPSs, PeSPP and PeINVs at three fruit development stages (T1, collected two weeks prior to harvest; T2, at the time of harvest; T3, one week after harvest) [40], were retrieved from the previously published RNA-seq datasets. For the abiotic stress treatments, healthy and virus-free passion fruit seedlings of purple fruit varieties (TN) with 8–10 surviving functional leaves (2–3 months old) were selected. The four abiotic stress treatments included: (1) drought stress - withholding water to achieve soil moisture levels of 50% (8 days after stopping watering) and 10% (13 days after stopping watering); (2) salt stress treatment - watering passion fruit plants with a solution containing 300 mM NaCl for 3 and 10 days; (3) low-temperature stress treatments - incubating passion fruit plants at 4 °C for 20 and 48 h; (4) high-temperature stress treatment - exposing passion fruit plants to a temperature of 42 °C for durations of 2, 4, and 24 h. The normalized expression data were utilized to generate a heatmap using TBtools.

In order to validate the expression patterns of sugar metabolism genes during fruit development and ripeness stages, four representative members with high relative expression levels were selected for qRT-PCR verification using the same samples employed in RNA-seq analysis. In addition, the expression levels of *PeCWINV5* in eight tissues (stem, leaf, root, petal, stamen, pistil, immature fruit and mature fruit) were also detected by qRT-PCR on a Roche fluorescence PCR (LightCycler 480II) using Takara TB Green^®^ Premix Ex Taq™ II fluorescence quantitative PCR kit with three biological replicates. The extension factor EF-1α was employed as an internal reference gene to normalize the data. The primer sequences used are provided in Supplementary Table 9. Following quantitative PCR analysis, the relative expressions were determined using the 2^-ΔΔCt^ method.

### Correlation analysis of *PeCWINV5* gene expression, sugar content, and fruit ripeness

Three fruit samples (T1, collected two weeks prior to harvest; T2, at the time of harvest; T3, one week after harvest) from the purple passion fruit variety “TN” and the yellow passion fruit variety “HJ” were collected and stored in a -80℃ refrigerator for sugar content analysis. “TN” and “HJ” are the predominant passion fruit varieties in China, with “TN” exhibiting a sweet and sour taste while “HJ” being purely sweet. Subsequently, frozen pulp samples weighing 0.5 g were ground into powder using liquid nitrogen and homogenized in 3.0mL of ethanol (80%) at a temperature of 35 °C for extraction. Extraction was performed three times, and all three extracts were combined, followed by centrifugation at room temperature (8000 rpm for 10 min) and filtration through a 0.22 μm filter membrane. A filtered solution volume of 10µL was utilized for sugar analysis employing high-performance liquid chromatography (HPLC) (Agilent 1260, Agilent Technologies, Santa Clara, CA, USA). The experimental conditions include a CNW amino column (250 mm × 4.6 mm, 3 μm), mobile phase consisting of acetonitrile: water (75:25, V/V), flow rate set at 1mL/min, column temperature maintained at 30 °C, and sample size injected as 10 µl. Detection was performed using an evaporative light scattering detector (ELSD). The quantity of individual sugars was calculated using peak areas of standards, and individual sugar contents were determined based on peak areas obtained from standards. The results were expressed as mg∙g^− 1^FW. Total sugar content was calculated as summing up sucrose content, glucose content, and fructose content. Sweetness index (SI) = (1.00×glucose level) + (2.30× fructose level) + (1.35 × sucrose level) [63].

### Transient overexpression in passion fruit pulp

For the transient overexpression vector, the complete coding region of *PeCWINV5* was inserted into a pCAMBIA1304 vector (Cambia, Canberra, Australia) downstream of the CaMV 35 S promoter using the Seamless cloning Master Mix (BBI, Shanghai, China). After confirmation by DNA sequencing, the recombinant pCAMBIA1304-*PeCWINV5* plasmid was transformed into Agrobacterium tumefaciens EHA105 cells. pCAMBIA1304 plasmid transformed into EHA105 cells were used as control. The EHA105 cells were diluted to an OD_600_ of 0.60 in infiltration buffer (10mM 2-(N-Morpholino) ethane sulfonic acid, 10mM MgCl_2_, 200µM Acetosyringone) for injection [64]. One passion fruit was separated into two part; one was injected with EHA105 cells containing the recombination vector and the other part with control EHA105 cells. The biologically independent transformations were performed in triplicate. After incubation at 28 ° C for 3 days, the fruit pulp was used for sugar contents evaluation.

### Statistical analysis methods

The Pearson correlation analysis was conducted using IBM SPSS 26 software. Statistical analyses, including the 2-way ANOVA analysis and multiple comparisons, were performed using GraphPad Prism 8 software. Each sample was replicated three times, and statistical significance levels were denoted as *, **, ***, and **** for *p* < 0.1, *p* < 0.01, and *p* < 0.001, respectively.

## Electronic supplementary material

Below is the link to the electronic supplementary material.

Supplementary Material 1

## Data Availability

The datasets generated and/or analyzed during the current study are available in the National Genomics Data Center (NGDC) repository, https://ngdc.cncb.ac.cn/search/?dbId=gwh&q=GWHAZTM00000000.
